# Determination of thermal sensation levels for Koreans based on perceived temperature and climate chamber experiments with hot and humid settings

**DOI:** 10.1007/s00484-022-02261-x

**Published:** 2022-03-04

**Authors:** Misun Kang, Kyu Rang Kim, Joo-Young Lee, Ju-Young Shin

**Affiliations:** 1 Operational Systems Development Department, National Institute of Meteorological Sciences, Seogwipo-si, Republic of Korea; 2 High Impact Weather Research Department, National Institute of Meteorological Sciences, Seogwipo-si, Republic of Korea; 3 Department of Textiles, Merchandising and Fashion Design, College of Human Ecology, Seoul National University, Seoul, Republic of Korea

**Keywords:** Thermal sensation vote, Predicted mean vote, Thermal perception, Heat resistance, Thermal stress

## Abstract

People perceive thermal sensation differently despite the same temperature value of thermal comfort index depending on various factors such as climate, culture, and physiological characteristics. The use of the thermal comfort index without optimization may lead to biases in assessment of thermal stress and sensation. This study aims to derive the perceived temperature (PT) ranges of thermal sensation levels related to heat stress for Koreans. The experiments were designed using a controlled environmental chamber to derive the PT ranges and were performed with subjects who are residents of Seoul, South Korea. The experiments were carried out in the summers of 2017 and 2018, and the thermal sensation votes were surveyed from 19 subjects whose mean age, height, weight, and body mass index were 22.5 years, 171 cm, 72 kg, and 23 kg⋅m^−2^, respectively. The derived PT ranges for Koreans led to a better performance than the reference PT ranges for Germans based on the results of validation. The thresholds of ‘Warm,’ ‘Hot,’ and ‘Very hot’ thermal sensation classes for Koreans were 28 °C, 36 °C, and 43 °C, respectively: higher than those for Germans. The results indicate that Koreans may have higher heat resistance or lower heat sensitivity than Germans.

**Supplementary Information:**

The online version contains supplementary material available at 10.1007/s00484-022-02261-x.

## Introduction

Thermal comfort can be explained as the balance of energy exchange between the human body and its environment (Gosling et al. 2014). Human thermal comfort is influenced by environmental factors such as temperature, humidity, wind speed, solar radiation, and mean radiant temperature (Budd 2008; Matzarakis et al. 1999). Additionally, human factors such as age, gender, physical activity, metabolic rate, and clothing are strongly associated with human thermal comfort (Nakano et al. 2002; Karjalainen 2012; Wang et al. 2018; Parsons 1999; Maykot et al. 2018). The use of various thermal comfort indices has been suggested, including perceived temperature (PT), physiological equivalent temperature (PET), and universal thermal climate index (UTCI), which are based on heat exchange between the human body and its thermal environment to evaluate biometeorological conditions (Jendritzky et al. 2012; Staiger et al. 2012, 2019; Höppe 1999). Since the thermal comfort index can take into account various factors in the assessment of thermal stress and sensation, it is swiftly becoming a mainstream metric of thermal environment assessment in scientific communities (Zhao et al. 2021). Use of the thermal comfort index can provide meaningful information for addressing economic and social issues including tourism (Rozbicka and Rozbicki 2020), planning of cities and urban design (Jänicke et al. 2019; Johansson and Emmanuel 2006), energy conservation (Yang et al. 2014), and public health (Urban and Kyselý 2014).

People perceive thermal sensation differently despite the same temperature value of thermal comfort index because thermal sensation varies with various factors such as climate, culture, and physiological characteristics (Lai et al. 2020; Cao et al. 2016; Jiang et al. 2019). Potchter et al. (2018) investigated the outdoor human thermal sensation in various climates and found that ‘Neutral’ sensation varies with the climatic condition in the region of interest. He et al. (2020) investigated cross-cultural thermal comfort differences in open spaces in a campus in Xi’an, China, for Chinese and Pakistani subjects. The ranges of ‘Neutral’ thermal sensations for Chinese and Pakistani subjects are different, as are their preferred actions for managing thermal discomfort. Kenawy and Elkadi (2021) examined the effects of cultural diversity on outdoor thermal perception in Melbourne, Australia. They reported that variations in thermal sensation votes are significantly related to the cultural and climatic backgrounds of the user. Thus, thermal comfort indices should be optimized for the accurate assessment of thermal stress and sensation (Ndetto and Matzarakis 2017; Sharmin et al. 2019). Pantavou et al. (2018) compared thresholds of thermal sensations for the UTCI and PET in different climatic regions and found that the threshold derived for the regions of interest would lead to a more accurate prediction of thermal comfort. Jeong et al. (2016) derived the PET range of ‘Neutral’ thermal sensation for Koreans using field measurements and questionnaires. This study did not investigate the other thermal sensations related to heat and cold stresses. Additionally, as this study derived thermal sensation based on field surveys, it may be difficult to correlate a specific environmental condition with the obtained results. Thus, the thermal comfort index ranges of thermal sensation levels related to heat and cold stresses are still absent for Koreans.

The PT is one of the predicted mean vote (PMV)-based thermal comfort indices (Staiger et al. 2012). The PT was developed based on the thermal sensations of Germans; as thermal sensations vary with various factors, particularly climate and culture, the use of PT without optimization may lead to biases in the assessment of thermal stress and sensation for people who live in other countries. Hence, the PT ranges of thermal sensation levels need to be derived for Koreans before its application for the assessment of thermal stress. Kang et al. (2020) nonetheless reported that the use of PT led to a better performance than the use of air temperature and wet-bulb globe temperature in the assessment of heat-related health risk in South Korea. The derivation of PT ranges of thermal sensation levels related to heat stress would provide a more accurate assessment of thermal stress and sensation in South Korea.

This study aims to derive the PT ranges of thermal sensation levels related to heat stress for Koreans. The experiments were designed to derive the PT ranges using a controlled environmental chamber and were performed upon subjects who are residents of Seoul, South Korea. The experiments were carried out in the summers of 2017 and 2018, and the thermal sensation votes (TSV) were collected from the subjects through a survey. New PT ranges of thermal sensation levels related to heat stress have thus been derived based on the surveyed TSVs. To the best of our knowledge, this is the first study to derive PT ranges of thermal sensation levels for Koreans. This study thus expects to improve our understanding of Korean physiological property to heatwave events. Additionally, the derived PT ranges for Koreans can lead to an improvement in the assessment of heat-related stress and health risks in South Korea.

## Materials and methods

### Perceived temperature

The perceived temperature (PT) is a thermal comfort index and is designed to assess the thermal physiology of people and is based on the ‘Klima-Michel’ model (KMM), which is an energy balance model for humans (Jendritzky et al. 1990). The PT is defined as ‘the air temperature of a reference environment in which the thermal perception would be the same as in the actual environment’ (Staiger et al. 2012). In the KMM for PT model, the reference person is a 35-year-old male with a height of 1.75 m, a weight of 75 kg, and a body surface area of 1.9 m^2^, an internal heat production of 135 W⋅m^−2^ (the metabolic rate of the reference person walking on flat ground at a speed of 4 km⋅h^−1^). The heat balance equation in KMM for the human body proposed by ASHRAE (2001) is as follows:

1 $$\mathrm{M}-\mathrm{W}=\left({\mathrm{C}}_{\mathrm{sk}}+{\mathrm{R}}_{\mathrm{sk}}+{\mathrm{E}}_{\mathrm{sk}}\right)+\left({\mathrm{C}}_{\mathrm{res}}+{\mathrm{E}}_{\mathrm{res}}\right)+{\mathrm{S}}_{\mathrm{sk}}+{\mathrm{S}}_{\mathrm{cr}}$$

Heat production within the body is related to the activity of the person. The human body consumes energy at the metabolic rate (M) so that it can do mechanical work (W), and the remainder of the metabolic rate (M-W) is the heat. Most of the energy released is in terms of heat: the heat transfer can be done by convection (C), radiation (R), and evaporation (E) through the skin (sk) and the respiratory system (res). The remaining heat is stored (S) in the skin and the core (cr) at a certain rate. For heat balance of the body, the rate of heat storage is zero (${\mathrm{S}}_{\mathrm{sk}}=0$ and ${\mathrm{S}}_{\mathrm{cr}}=0$). When the internal heat production is identical to the heat, which is exchanged with the external environment at a steady state, the PMV can be expressed by Eq. (2).

2 $$\mathrm{PMV}=\mathrm{\alpha }\cdot \left\{\mathrm{M}-\mathrm{W}-\left({\mathrm{C}}_{\mathrm{sk}}+{\mathrm{R}}_{\mathrm{sk}}+{\mathrm{E}}_{\mathrm{sk}}\right)-\left({\mathrm{C}}_{\mathrm{res}}+{\mathrm{E}}_{\mathrm{res}}\right)\right\}$$

When calculating PT in KMM, the PMV equation modified by Gagge et al. (1986) was employed. Since the PMV accounts for energy exchange based on a two-node body model, latent and sensible heat transfer is from or to the skin (considering sweating) and by respiration. The PMV model includes main parameters influencing thermal sensation such as air temperature (Ta, °C), relative humidity (RH, %), wind speed (WS, m⋅s^−1^), mean radiant temperature (Tmrt, °C), activity level, and clothing insulation (I_cl_, clo). The activity level can be considered as a function of metabolic rate. The PT contains a clothing model, which automatically adapt to hot or cold conditions with a clothing insulation range between I_cl_ = 1.75 clo (cold) and I_cl_ = 0.50 clo (hot). In the heat stress condition where PMV is greater than zero, the PT is calculated by the following equation:

3 $$\mathrm{PT}=6.18\bullet \mathrm{PMV}+16.83$$

Detailed information upon the calculation of PT can be found in Staiger et al. (2012).

**Fig. 1:**
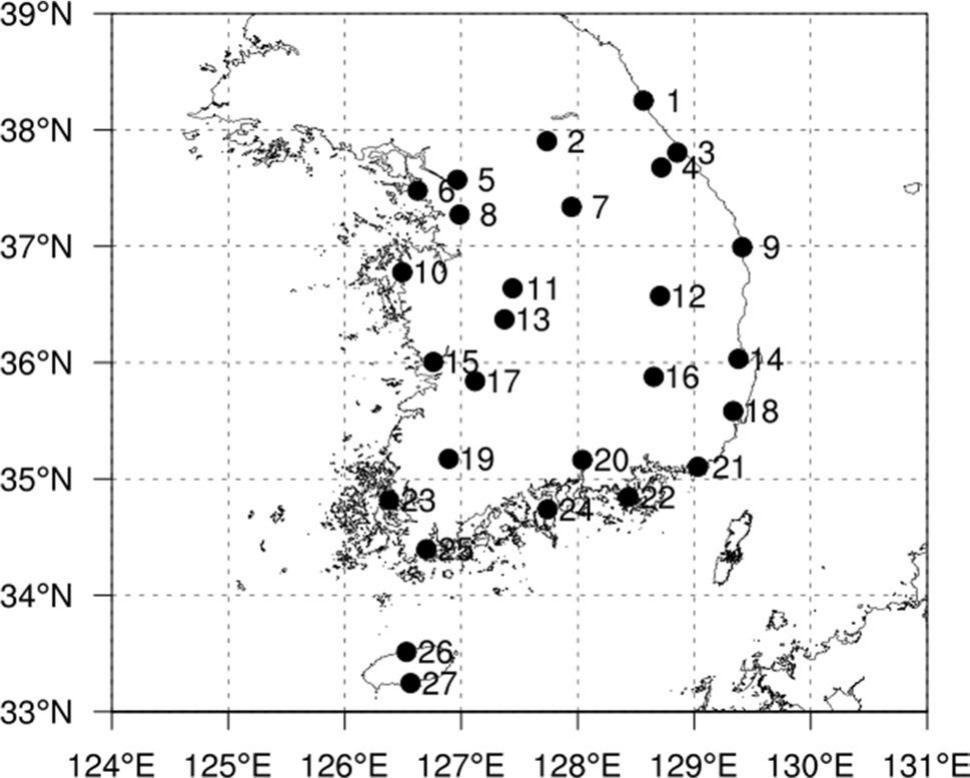
Geographical locations of the employed stations. Detailed information of the employed 27 weather stations is presented in Table S1 of the supplementary information

In the present study, the PT inside the chamber was calculated using experimental environmental conditions, which are Ta, RH, WS, and the metabolic rates of subjects. When calculating PT inside the chamber, Tmrt was assumed to be equal to Ta. Additionally, the outside PT was calculated using data, which are the Ta, RH, WS, cloud amount, cloud type, and geographical information. The data were obtained from 27 meteorological stations of the Korea Meteorological Administration (KMA). The outside Tmrt was calculated using cloudiness information because measurements of solar radiation are conducted only at a few meteorological stations in KMA. The meteorological data cover summer months: June, July, August, and September over a 30-year period (from 1990 to 2019). The hourly PT values were calculated using hourly observed meteorological parameters, and the daily maximum PT values were collected to compare the proportions of thermal sensation classes by PT ranges. Details about the meteorological stations are presented in Fig. 1 and supplementary information.

### Experimental site and subjects

**Fig. 2:**
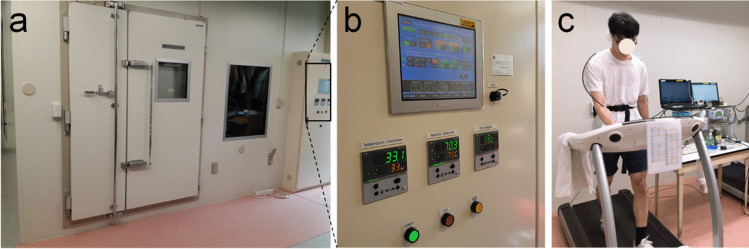
**a** Controlled environmental chamber, **b** temperature and relative humidity controller, and **c** a subject in the experiments

In this study, all experiments were conducted in a controlled environmental chamber which has the capability to control ambient temperature with ± 0.5 °C, and relative humidity with ± 3%, of the Seoul National University in Seoul, South Korea. The chamber is a 6.5 m × 3.6 m × 2.8 m (width × length × height) room with external wall, door, and window (Fuji Medical Science, Japan) and is shown in Fig. 2. Seoul is located at 37.45°N and 126.95°E (NO. 5 in Fig. 1) and has hot and humid climate in the summer, which lasts from July to August. Over the summer of 30 years from 1990 to 2019, the mean and maximum Ta and mean RH were 25.7 and 34.6 °C and 74.9%, respectively.

The experimental subjects were 11 and 9 young males who lived in Seoul in 2017 and 2018, respectively. They were healthy, i.e., not taking prescription medications and without cardiovascular or endocrine system diseases. The mean body mass parameters, such as age, height, weight, and body mass index (BMI) of the subjects in the 2017 experiment, were 23.5 ± 2.3 years, 167.2 ± 7.0 cm, 73.7 ± 10.0 kg, and 23.9 ± 2.3 kg⋅m^−2^, respectively; for the subjects in the 2018 experiment, the mean body mass parameters were 21.3 ± 2.6 years, 175.2 ± 4.0 cm, 70.1 ± 7.9 kg, and 22.8 ± 2.2 kg⋅m^−2^, respectively. The mean of the theoretical metabolic rates for all subjects was 137.8 W⋅m^–2^ (Jendritzky et al. 1990), and the difference between the metabolic rate of the reference person in the KMM and the mean of theoretical metabolic rate was very small. This fact supports the hypothesis that experimental results based on these subjects may have a similar thermal sense to that used in the development of PT. All subjects were sufficiently informed about the experimental purpose, experimental procedure, and measurements, and then they agreed to participate in the study. To attenuate any influences from human circadian rhythms for individual subjects, each subject participated in the experiment once a day from 09:00 to 12:00 Korea standard time (KST). Additionally, the experimental procedures were kept exactly the same for all the experiments, so as to ensure the comparability of the results. The subjects were asked to avoid caffeine, alcohol, and intense physical activity for at least 12 h prior to the experiment and were instructed to have breakfast at least 2 h prior to the experiment. Before the experiment, the subjects were asked to drink mineral water (300 mL) to avoid becoming dehydrated and to change their clothes to the uniform clothing provided by the researchers.

### Experimental design and conditions

To assess the heat stress of Koreans, the experiments were carried out in the summers (June to August) of 2017 and 2018. The total experimental time of one trial for one subject was 70 min, which included 10 min for rest and 60 min for activity. For the experiment, subjects were required to walk at 4 km⋅h^–1^ on flat, and clothing condition was 0.4–0.5 clo, including short-sleeved t-shirts, short pants, underpants, socks, and sneakers as summer clothes. Additionally, the environmental conditions were set to simulate the summer climate in Korea, i.e., hot and humid atmosphere. In the chamber, the Ta was set to 30 °C and 35 °C in 2017 and 28 °C, 33 °C, and 38 °C in 2018, respectively. The RH was 70% and WS was lower than 0.005 m⋅s^–1^. The environmental conditions in the chamber were constant during the test period. Based on the experimental designs, PT values were 35 °C and 44 °C in 2017, and 31 °C, 40 °C, and 49 °C in 2018.

**Fig. 3:**
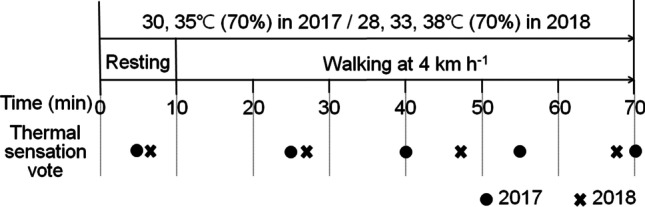
Tabular representation of the experimental procedure. Circle and cross markers indicate time to obtain subject response for experiments in 2017 and 2018, respectively

A psychological questionnaire was carried out to investigate the actual thermal feeling of subjects during the experiments. The questionnaire was designed to reflect the respondents’ subjective assessment of the thermal environment. The subjects were required to report subjective responses, namely, the TSV. The term thermal sensation refers to the human thermal sense (feeling hot or cold, etc.). Considering the hot and humid conditions, a 9-point extended scale from ISO 10551 (ISO 1995) was used for the TSV. The scale of TSV used in this study is listed in Table 1. All experiments in 2017 and 2018 were set to the same experimental procedure, measurements, activity, and clothing conditions except for Ta in the environmental condition. The experimental process is briefly summarized in Fig. 3.

**Table 1:** Scales of thermal sensation vote

TSV class	Thermal sensation level
Very hot	4
Hot	3
Warm	2
Slightly warm	1
Neutral	0
Slightly cool	-1
Cool	-2
Cold	-3
Very cold	-4

### Derivation of PT ranges of thermal sensation levels for Koreans

The PT ranges of thermal sensation levels for Koreans were derived based on TSV that refers to responses for TSV from subjects in the 2017 experiment. There were exceptional heatwave events in the northern hemisphere as well as in South Korea in the summer of 2018 (Yiou et al. 2020; Larcom et al. 2019; Min et al. 2020; Im et al. 2019). Due to the fact that experiencing exceptional heat stress will lead to additional thermal adaptation, use of the 2018 data in deriving the PT ranges could lead to large biases. Therefore, data of the experiment in 2018 were only used to evaluate the appropriateness of the derived PT ranges. Thermal sensations differ among individuals even when they are in the same environment (Lin and Matzarakis 2008). Additionally, the mean Ta (25.4 °C) during the summer of 2017 in Seoul was very close to the mean Ta (25.3 °C) of summer over the 10 years (from 1999 to 2018) and corresponded to 54.5% in the distribution of the mean Ta of summer in Seoul. The mean Ta (26.6 °C) during the summer of 2018 in Seoul is much higher than the mean Ta of summer during the last 10 years, i.e., 95.1%. Since there is a strong warming signal in Ta in South Korea (Shin et al. 2021), the Ta data during summer in the last 10 years were used in the calculation of the mean Ta to avoid global warming effects. Therefore, the mean TSV for each PT level was used in order to obtain the representativeness of the PT ranges. The linear regression model using mean TSV as the dependent variable and PT as the independent variable was fitted based on the data from the experiment. Finally, thermal sensation levels for Koreans were derived based on the fit linear regression model. The PT ranges of thermal sensations were considered to be thermal stress levels. In this study, the PT levels were optimized for heat stress of summer in South Korea.

The appropriateness of the new PT ranges of thermal sensation levels for assessing heat stress for Koreans was evaluated using the TSV from the subjects of the 2018 experiment. For comparison, the appropriateness of the reference PT ranges was also evaluated. The precision (P), recall (R), F1 score (F), and accuracy (Acc) based on a confusion matrix with 5 classes were used as evaluation criteria. The confusion matrix in this study is two dimensions (class i and j) and contains information about actual (class i) and predicted (class j) classes which are divided from Class 0 to Class 4. Class 0 to 4 indicate ‘Neutral’ to ‘Very hot’ thermal sensation levels, respectively. In this study, class *i* is TSV responses results that are obtained from the subjects in the 2018 experiment. The class *j*, namely predicted TSVs, is thermal sensation levels classified by reference and derived PT range in the 2018 experimental environment (PT 31 °C, 40 °C, and 49 °C), respectively. In the confusion matrix, the total number of false negatives (*TFN*), false positives (*TFP*), true negatives (*TTN*), and true positives (*TTP*) for each class *i* can be calculated using Eqs. (4)–(7):

4 $${TFN}_{i}= {\sum }_{\begin{array}{c}j=0\\ j\ne i\end{array}}^{4}{x}_{ij}$$

5 $${TFP}_{i}= {\sum }_{\begin{array}{c}j=0\\ j\ne i\end{array}}^{4}{x}_{ji}$$

6 $${TTN}_{i}= \sum\nolimits_{\begin{array}{c}j=0\\ j\ne i\end{array}}^{4}\sum\nolimits_{\begin{array}{c}k=0\\ k\ne i\end{array}}^{4}{x}_{jk}$$

7 $${TTP}_{i}= {\sum }_{i=0}^{4}{x}_{ii}$$

where ${x}_{ij}$ is the number of scores corresponding to class *i* for predicted TSV and class *j* for TSV. TFN means a case in which the predicted TSV incorrectly predicts the positive TSV, and TFP means a case in which the predicted TSV incorrectly predicts the negative TSV. Also, TTN means a case in which the predicted TSV correctly predicts the negative TSV, and TTP means a case in which the predicted TSV correctly predicts the positive TSV. The *P*, *R*, *F,* and *Acc* for each class *i* can be calculated by following Eqs. (8)–(11):

8 $${P}_{i}= \frac{{TTP}_{i}}{{TTP}_{i}+{TFP}_{i}}$$

9 $${R}_{i}= \frac{{TTP}_{i}}{{TTP}_{i}+{TFN}_{i}}$$

10 $${F}_{i}= 2\times \frac{{P}_{i}\times {R}_{i}}{{P}_{i}+{R}_{i}}$$

11 $$Ac{c}_{i}= \frac{{TTP}_{i}+{TTN}_{i}}{Total number of data}$$

## Results

### PT ranges of thermal sensation levels for Koreans

**Fig. 4:**
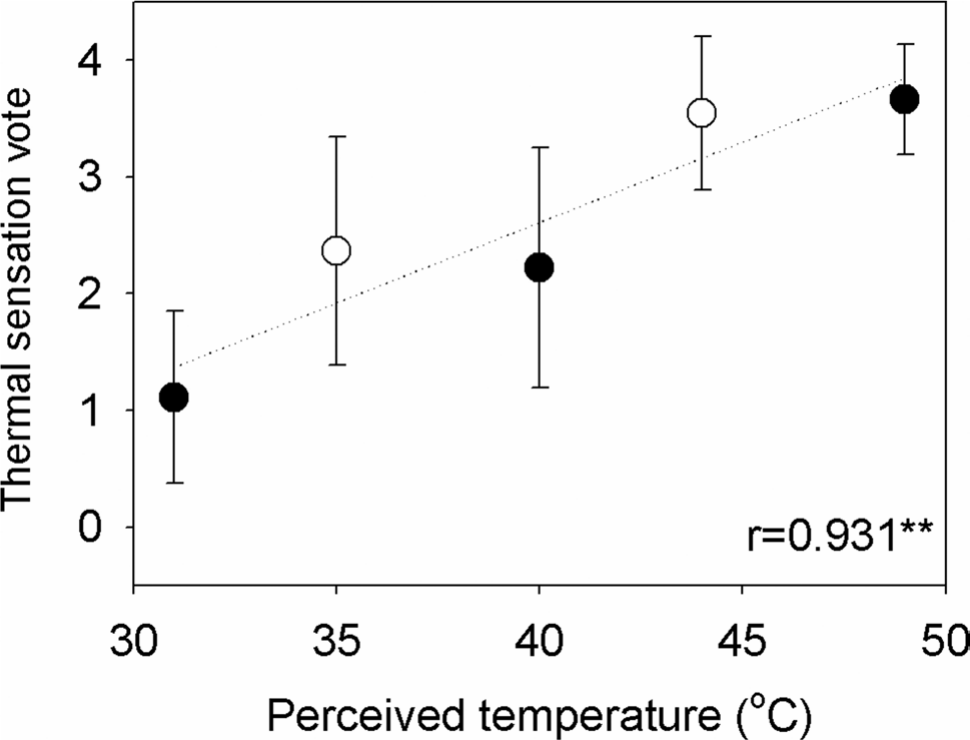
The relationship between perceived temperature (PT) and the subjective responses of thermal sensation vote (TSV) for the experimental conditions of the 2017 (*open circle*) and 2018 (*closed circle*). The circle and bar indicate the mean and standard deviation, respectively, of the subjective responses of the subjects in each experimental condition. The PT of the experimental conditions correlated with the mean subjective responses. Note that ** indicates that the Pearson correlation (r) estimates are significant with 5% significance level

**Fig. 5:**
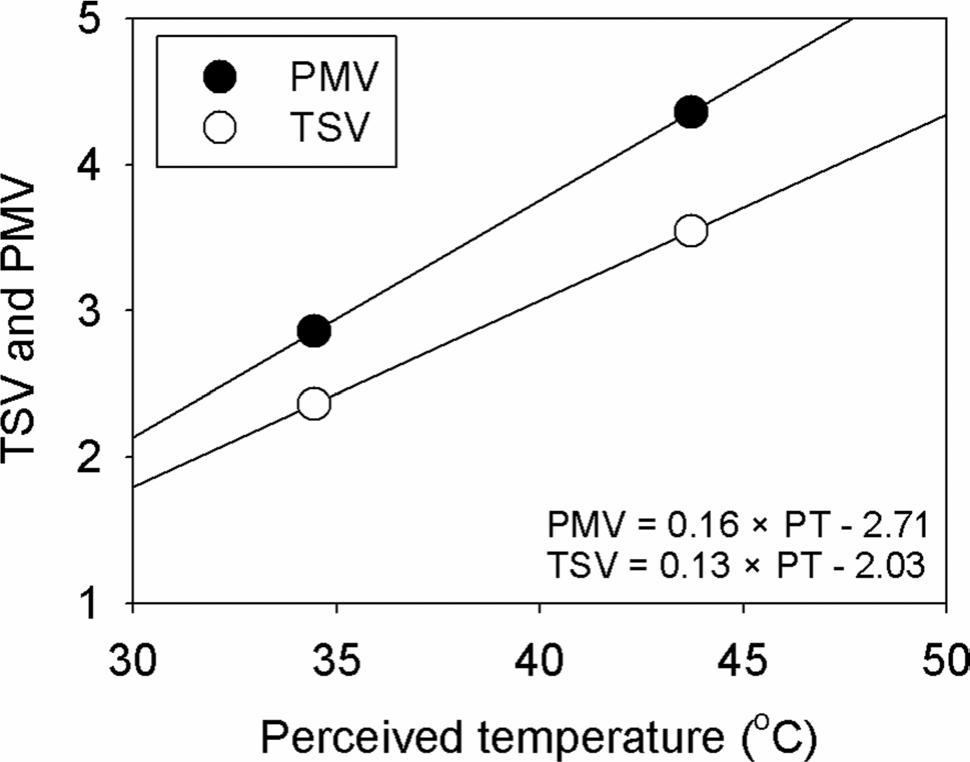
Relationship between TSV and PT (TSV = 0.13 × PT-2.03) and between PMV and PT (PMV = 0.16 × PT-2.71). Open circles indicate the TSV that refers to responses for TSV from subjects in the 2017 experiment, and closed circles indicate the original PMV thermal sensation classes for the reference of the experiments

The relationship between PT and the mean subjective responses for each experimental condition of 2017 and 2018 is shown in Fig. 4. The result shows the mean and standard deviation (SD) of each subjective response investigated at 55 min. The closed and open circles indicate the subjective responses of TSV for the 2017 and 2018 experimental conditions, respectively. The TSV has a positive relationship with PT. The values of Pearson’s correlation coefficient were 0.931 between TSV and PT and were statistically significant with 5% significance level. The mean TSV increased from 1.11 to 3.67 with the increase of the PT. Above 40 °C, subjects responded that TSV was ‘hot’ or ‘very hot.’ The results show that each subjective response varied with PT and subjects felt hotter with increasing PT. The relationships between PT and TSV, and between PT and the PMV from the 2017 experiment are illustrated in Fig. 5. A linear regression model was employed to describe the relationship between PT and TSV. The linear regression model was fitted using data of the 2017 experiment and is presented in Eq. (12).

12 $$\mathrm{TSV}=0.13\times \mathrm{PT}-2.03$$

It can be assumed that the TSV from the subjects in this experiment represented the thermal sensations of Koreans. At the same values of PT, TSV was smaller than PMV, and the slope of the relationship between PT and TSV was smaller than that between PT and PMV (Fig. 5). As the PT increased by 1 °C, the TSV increased by 0.13. Since the PT range of thermo-physiological stress levels is equivalent to the PT range of thermal sensation, the derived PT ranges of thermal sensations were used as the PT ranges of thermal sensation classes for Koreans. The PT ranges of thermal sensation levels for Germans (the reference PT ranges) and Koreans are presented in Table 2.

**Table 2:** Perceived temperature ranges of thermal sensation class and thermo-physiological stress level for Germans and Koreans

Thermal sensation class	PT (Germans, °C)	PT (Koreans, °C)	Thermo-physiological stress level
Very hot	PT ≥ 38	PT ≥ 43	Extreme heat stress
Hot	32 ≤ PT < 38	36 ≤ PT < 43	Great heat stress
Warm	26 ≤ PT < 32	28 ≤ PT < 36	Moderate heat stress
Slightly warm	20 ≤ PT < 26	20 ≤ PT < 28	Slight heat stress
Comfortable	0 ≤ PT < 20	PT < 20	Comfort possible

The threshold of the ‘Very hot’ thermal sensation for Koreans was 43 °C and was larger than that (38 °C) for Germans. The thresholds for the ‘Hot’ and ‘Warm’ thermal sensations for Koreans were 36 °C and 28 °C, respectively. These values were also higher than those for Germans (32 °C and 26 °C, respectively). The range extents of ‘warm’ and ‘hot’ thermal sensations for Koreans were 8 °C and 7 °C, respectively, and were likewise larger than those for Germans (6 °C). The threshold of the ‘Slightly warm’ thermal sensation was 20 °C for both; however, the range extents for Koreans were broader than those for Germans. In summary, the thresholds of thermal sensation classes related to heat stress and the range extents were higher and wider, respectively, for Koreans than those for Germans. These results indicate that Koreans have higher thermal tolerance or lower thermal sensitivity than Germans in hot and humid environments.

### Comparison of the reference and Korean PT ranges of thermal sensation classes using experimental data in 2018

The appropriateness of the new PT ranges of thermal sensation levels for assessing heat stress for Koreans was evaluated using the TSV from subjects in the 2018 experiment. For comparison, the appropriateness of the reference PT ranges was also evaluated. The new PT ranges are the derived PT range based on TSV from the subjects in the 2017 experiment, and the reference PT ranges are the original PT ranges for Germans (see Table 2). The confusion matrices for the reference and Korean PT ranges using the experiment data in 2018 are presented in Table 3. The observed TSV in Table 3 is the subjects' responses from the 2018 experiment, whereas the predicted TSVs are thermal sensation levels based on the reference and Korean scales. For example, if a subject responded 'Slightly warm' at the experiment of PT 40 °C, observed TSV is classified as Class 1 (Slightly warm), while the predicted TSVs are classified as Class 4 (Very Hot) or Class 3 (Hot) by reference scale or Korean scale, respectively. *P*, *R*, *F*, and *Acc* for each class are listed in Table 4. The results indicated that the Korean PT ranges were more appropriate than the reference PT ranges in the assessment of thermal sensation level for Koreans in 2018. The Korean PT ranges performed better based on the chosen evaluation measures, except *P*. Although the average *P* of the reference PT ranges is 0.73, and this value is higher than that (0.71) of the Korean PT ranges, the latter provided higher *P* values than the reference for assessing the ‘Very hot’ thermal sensation class, which is equivalent to the ‘Extreme heat stress’ class in thermo-physiological stress levels and is important in assessing heat-related health risks. Overall, the average *Acc* of the Korean PT ranges is 0.8 and is higher than that (0.71) of the reference PT ranges. Large differences between observed TSV and predicted TSV for the reference PT ranges were observed in the ‘Hot’ and ‘Very hot’ thermal sensation classes. When the observed TSV indicated ‘Hot,’ the reference PT ranges predicted the ‘Very hot’ thermal sensation class for all subject responses. This result implies that thermal sensation classes are overestimated by the reference PT ranges for the ‘Hot’ thermal sensation class. Unlike the reference PT ranges, use of the Korean PT ranges improves accuracy in predicting thermal sensation classes for ‘Hot’ and ‘Very hot.’ For instance, *F* increased from 0 to 0.59 for the ‘Hot’ thermal sensation class and from 0.5 to 0.8 for the ‘Very hot’ thermal sensation class by using the Korean PT ranges.

**Table 3:** Confusion matrix between observed TSV and predicted TSVs based on the reference and derived PT ranges of thermal sensations. Classes 0 to 4 indicate ‘Neutral’ to ‘Very hot’ thermal sensation levels, respectively. TSV indicates responses results obtained from the subjects in the 2018 experiment, and the predicted TSVs indicate thermal sensation levels classified by reference and derived PT range based on the 2018 experimental environment (PT 31 °C, 40 °C, and 49 °C), respectively. Bold face indicates true positive cases

Observed TSV		Predicted TSV									
		by reference (German) PT range					by derived (Korean) PT range				
		Class 0	Class 1	Class 2	Class 3	Class 4	Class 0	Class 1	Class 2	Class 3	Class 4
	Class 0	**0**	0	2	0	1	**0**	0	2	1	0
	Class 1	0	**0**	4	0	1	0	**0**	4	1	0
	Class 2	0	0	**3**	0	2	0	0	**3**	2	0
	Class 3	0	0	0	**0**	8	0	0	0	**5**	3
	Class 4	0	0	0	0	**6**	0	0	0	0	**6**

**Table 4:** Evaluation results of the reference and derived PT ranges

	Version	Neutral	Slightly warm	Warm	Hot	Very hot	AVG
Precision	Reference	1.00	1.00	0.33	1.00	0.33	0.73
	Derived	1.00	1.00	0.33	0.56	0.67	0.71
Recall	Reference	0.00	0.00	0.60	0.00	1.00	0.32
	Derived	0.00	0.00	0.60	0.63	1.00	0.45
F1 Score	Reference	0.00	0.00	0.43	0.00	0.50	0.19
	Derived	0.00	0.00	0.43	0.59	0.80	0.36
Accuracy	Reference	0.89	0.79	0.68	0.56	0.71	0.71
	Derived	0.89	0.79	0.68	0.74	0.89	0.80

### Comparison of proportions of thermal sensation classes obtained by the reference and derived PT ranges in meteorological conditions during summer

**Fig. 6:**
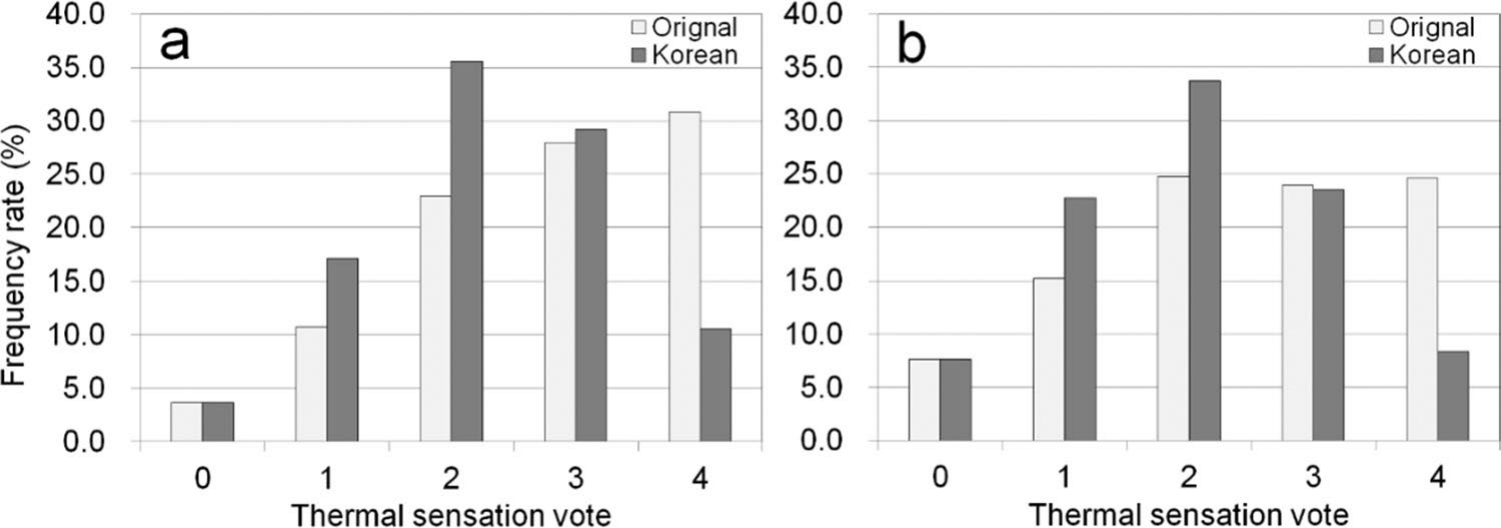
The frequency rates distribution of thermal sensation classes using reference and Korean PT ranges for daily maximum PT (**a**) from June to September and (**b**) from May to September. Note that the daily maximum PT data covered over a 30-year period (from 1990 to 2019) of 27 meteorological stations of KMA, and classes 0, 1, 2, 3, and 4 indicate ‘Neutral,’ ‘Slightly warm,’ ‘Warm,’ ‘Hot,’ and ‘Very hot’ thermal sensations, respectively

The frequency rates of each thermal sensation class based on the reference and Korean PT ranges for daily maximum PT in South Korea are presented in Fig. 6. The data of daily maximum PT covered summer months: June to September or May to September during 30 years (from 1990 to 2019) of 27 meteorological stations of KMA (see Fig. 1). Although summer in South Korea is generally considered June to September, the period from May to September is also accounted for due to the global warming that has led to increased number of heat-related illness cases in May. Also, although the experiments were conducted in the chamber located in Seoul, the derived PT range was applied across the country in South Korea as the PT range in the experiments represented the whole range of Korean summer climate. For the period from June to September, the highest value (30.8%) reached the ‘Very hot’ thermal sensation class based on reference PT ranges followed by ‘Hot’ (27.9%), ‘Warm’ (23.0%), ‘Slightly warm’ (10.8%), and ‘Neutral’ (3.7%). For the Korean PT ranges, the highest frequency rate (35.6%) was of the ‘Warm’ thermal sensation class. The frequency rates of certain thermal sensations decreased as the difference from the ‘Warm’ thermal sensation class increased. The reference PT ranges led to overestimation of the ‘Very hot’ thermal sensation class as compared to the Korean PT ranges, while also leading to a large underestimation for the ‘Warm’ thermal sensation class. For the period starting May, although the frequency rate of the ‘Warm’ thermal sensation class had the highest value for the reference PT ranges, the frequency rate of the ‘Very hot’ thermal sensation class was still high. In the case of the Korean PT range, the ‘Warm’ thermal sensation class had the highest frequency rate. The frequency rates of ‘Warm’ and ‘Very Hot’ thermal sensation classes were higher and lower than those from June to September, respectively. The distribution of thermal sensations based on reference PT ranges had a positively skewed shape, while the Korean PT ranges had a bell shape distribution.

**Fig. 7:**
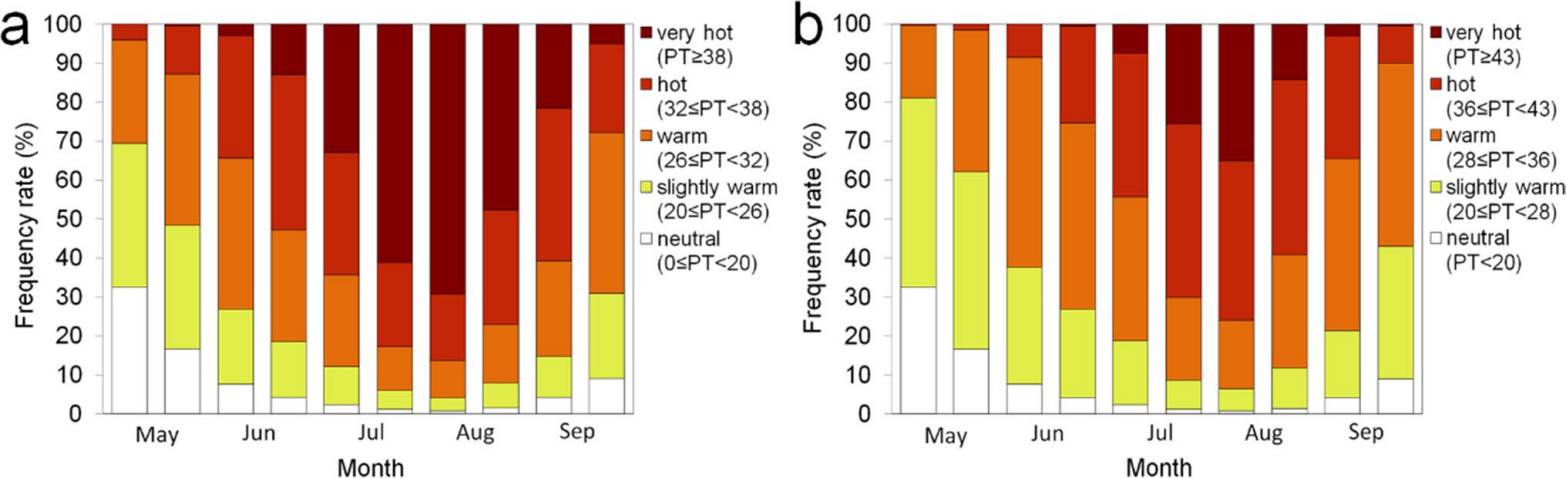
The frequency rates of thermal sensation classes using (**a**) reference and (**b**) Korean PT ranges for daily maximum PT from May to September for 1990–2019 of 27 meteorological stations of KMA and presented in 15-day units

In order to investigate the changes in frequency of thermal sensation classes during summer, the frequency rates of thermal sensations classes based on daily maximum PT, in 15-day intervals from May to September, were calculated (Fig. 7). There are discrepancies between the frequency rates of thermal sensation classes. The frequency rates of ‘Very hot’ and ‘Hot’ had high values in July and August for both the reference and Korean PT ranges; however, their values from the reference and Korean PT ranges were different. For the reference PT ranges, ‘Very hot’ appeared from early June to late September, and the proportions in late July and early August were 61.2% and 69.2%, respectively. In contrast, ‘Very hot’ appeared from early July to early September based on the Korean PT ranges, and the proportions in late July and early August were 26.1% and 33.9%, respectively. In summary, the frequency rates for ‘Warm’ and ‘Hot’ thermal sensation classes were higher than those for reference PT ranges. The results imply that the reference PT ranges tend to overestimate thermal sensation classes related to heat stress in South Korea.

## Discussion

**Fig. 8:**
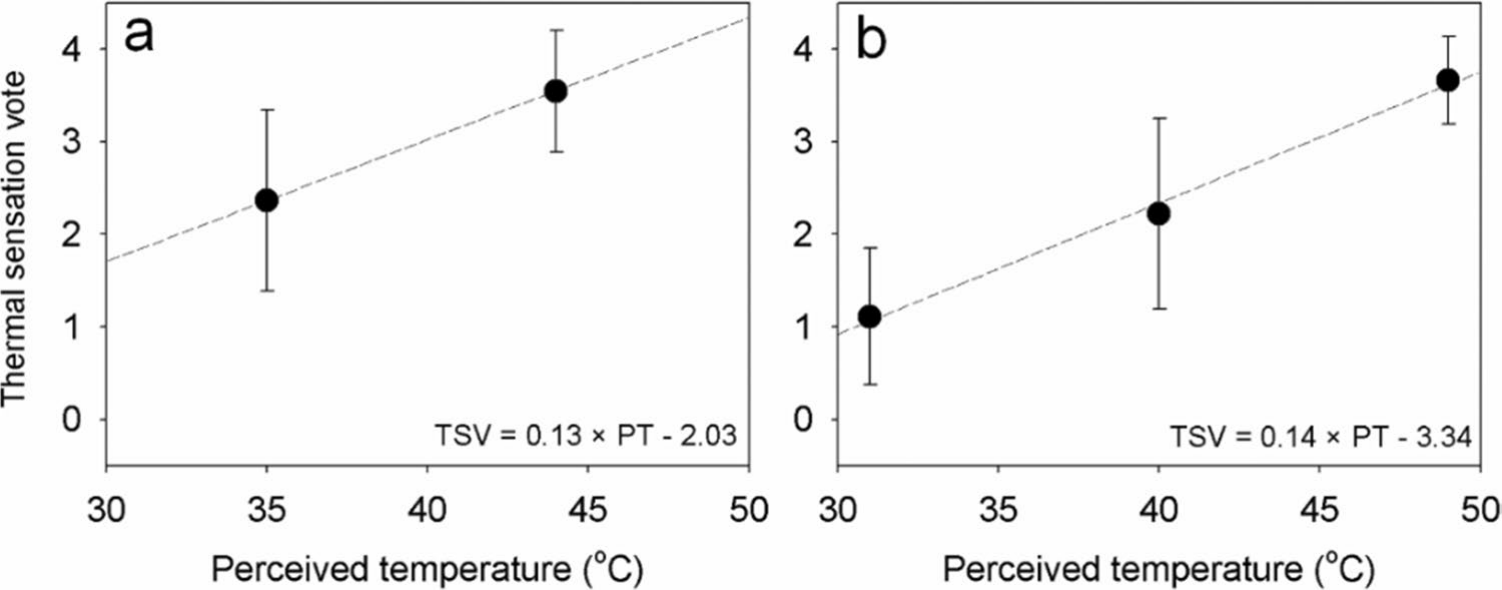
The relationship between perceived temperature (PT) and the subjective responses of thermal sensation vote (TSV) for the experimental conditions of the (**a**) 2017 and (**b**) 2018. The black circle and bar indicate the mean and standard deviation, respectively, of the subjective responses of the subjects in each experimental condition. The PT of the experimental conditions correlated with the mean subjective responses

Figure 8 shows the relationship between PT and the mean subjective responses for each experimental condition of 2017 and 2018, respectively. Results of experiments in 2017 and 2018 showed that prior experience of the subject to thermal environment influences on the subjective responses (also see Fig. 4). The environmental conditions of the experiment (PT = 31 °C, 41 °C, and 49 °C) in 2018 reached higher PT values than those (PT = 35 °C and 44 °C) in 2017. The observed TSV showed positive relationships with PT in both experiments. The slope of the relationship between PT and TSV in the 2017 experiment was similar to that in the 2018 experiment. As the PT increased by 1 °C, the TSV from the experiment in 2017 and 2018 increased by 0.13 and 0.14, respectively (see Fig. 8). However, the Y-intercepts in 2017 and 2018 were -0.23 and -3.34, respectively, and the TSVs from the experiment in 2018 seem to be approximately 1 smaller than those from the experiment in 2017 at the same PT level. As a matter of fact, exceptional heatwave events occurred in South Korea in the summer of 2018 (Im et al. 2019; Min et al. 2020), with the record long (31.5 days) heat wave and all-time high (38.4 °C) temperature since the beginning of KMA observation in Seoul. Earlier studies reported that heat acclimation begins on the initial day of exposure and is complete after 7–10 days or 2 weeks (Eichna et al. 1945; Sawka et al. 1996; Pandolf 1998). The prior experience of the subjects to exceptional heatwave events led to their heat acclimation in 2018. Because this heat acclimation may have affected the heat resistance of the subjects, the TSVs from the subject responses in 2018 were smaller than those in 2017 at the same PT levels. Thus, the appropriate choice was to exclude the data in 2018 from deriving the PT ranges for Koreans. If the experimental data in 2018 were used, the derived PT ranges may have had large biases and might not accurately represent the thermal sensations of Koreans in normal conditions.

In this study, the experimental data in 2018 were instead used to evaluate the appropriateness of the Korean PT ranges. The results showed that the Korean PT ranges derived from the experimental data in 2017 provided a robust performance, despite external factors that could disturb the thermal perception of the subjects. This evidence supports the hypothesis that the Korean PT ranges in this study can be used to assess thermal sensations of Koreans for extreme heatwave events.

The reference PT range currently used in Germany has been optimized using the fit linear regression model obtained from the relationship between PT and PMV (see Fig. 5). The PMV obtained using Eq. (2) represents thermal sensations for Germans, because this model was developed based on the physiological characteristics of Germans. The relationship between TSV and PT obtained by the chamber experiments in this study differs from the relationship between PT and PMV, which is not what would have been expected if the PMV equation successfully represents the thermal sensations for Koreans. The slope in the relationship between TSV and PT is lower than that in the relationship between PMV and PT (see Fig. 5). This difference leads to the difference of the PT range of thermal sensation classes related to heat stress for Germans and Koreans. The thresholds and the range extent for Koreans were higher and wider, respectively, than those for Germans (see Table 2). The results support the fact that the thermal sensations of Koreans and Germans are different. This difference may result from many factors: people’s thermal sensations are associated with their climate, culture, clothing insulation, and age (Nakano et al. 2002; Wang et al. 2018; He et al. 2020). Firstly, the age of the subject and the reference person of KMM (PT model) are different. TSV is surveyed from the male subjects of their 20 s, whereas PMV is calculated based on 35-year-old reference person. Previous studies showed different thermal sensation among age groups such as younger (their 20 s) and elderly (their 60-70 s) groups (Natsume et al. 1992; Wang et al. 2018). However, the difference in thermal sensation between those in their 20 s and 30 s could not be identified experimentally. Thus, it was assumed that there was no difference in the thermal sensation of their 20 s and 30 s in this study. In the case of the clothing insulation, as the clothing insulation (0.4–0.5 clo) of subjects in this study was similar to the clothing insulation (0.5 clo) for summer in the KMM (PT) model, so it should have not affected the difference between TSV and PMV. Lastly, summer in South Korea is hotter and more humid than in Germany. This high temperature and humid environment might lead to heat acclimation, which would eventually lead Koreans having higher heat resistance than Germans. Many studies focused on studying thermal comfort are based on Caucasian responses (Zhao et al. 2021). To expand the applicability of the thermal comfort index in other regions, the physiological characteristics for people in those regions should be investigated and the thermal comfort index should be modified according to the responses by the people of interest.

To reduce damages from the heat-related health risk and assess heat-related health risks for Koreans, it is critical to define appropriate thresholds of thermal sensation classes based on their physiological properties. Our experimental results showed the reference PT ranges overestimated the thermal sensation classes—particularly the ‘Hot’ thermal sensation class. These results lead to the inference that the reference PT ranges are inappropriate for assessing the thermal sensations of Koreans and bolster the need to derive new PT ranges. The derived PT ranges for Koreans provide a more accurate estimation of thermal sensations related to heat stress than the reference PT ranges. Therefore, the Korean PT ranges should be used in the assessment of heat-related health risks for Koreans to support decision and policy makers and in designing appropriate mitigation strategies, particularly for occupational health risks for outdoor workers.

To optimize the thermal sensations for people of interest based on the PT index, two approaches are possible. The first one is the modification of the PMV equation in the PT calculation, and the other is to derive new PT ranges for thermal sensations and stresses. The first approach changes the PT values and retains the PT ranges for thermal sensation classes and thermal stress levels. In contrast, the second approach of deriving new PT ranges retains the original PT values. The differences in PT values from the first approach could lead to confusion in the interpretation of the interactions between the thermal environment and the human body. By deriving new PT ranges, the physiological characteristics of the people of interest is taken into consideration in the assessment of thermal sensation and thermal stress. As a simple modification of the PT range may not fully represent physiological characteristics, the second approach may have a limited capacity to assess thermal stress. Nevertheless, because the PT is always a single value in a given environmental condition, the second approach can avoid confusion. Additionally, the PT values obtained by the second approach can be intercomparable among different environments. The PT index thus has the potential to be used as a universal index to measure the heat load of the environment to the human body. We thought that this property of PT would be important in the evaluation of its scientific robustness in the assessment of thermal stress and of the environment by intercomparing studies worldwide. Hence, this study adopted the second approach to develop a PT for Koreans in assessing thermal stress. The appropriateness of the two approaches should be discussed and examined in future research.

In the approach used in this study, the temperature calculated in the human thermal comfort or thermal stress model for human physiology (e.g., PT) is the same as the original model. In this study, the thermal stress level is compensated using the TSV-PT range relationship from the controlled environmental chamber experiments. The procedures for finding out a new PT range using observed TSV and onsite PT can be automated further by region, age, gender, or even individuals. The thermal sensation of any region and any group of people can be accurately evaluated in the end.

The vulnerability of people to heatwave events is strongly associated with age (Meade et al. 2020; Jiao et al. 2020; Ma et al. 2021). In particular, the elderly people are more vulnerable to heat-related health risks than young people at the same thermal exposure levels (Chen et al. 2015; Lee et al. 2018). Hence, a new PT model should be developed for different ages to assess heat-related health risks more accurately. Matzarakis et al. (2020) proposed the Klima-Michel Senior Model, which considers the physiological characteristics of elderly people in thermal stress assessments. It takes into account age-dependent changes in thermoregulation and a lower metabolic rate which corresponds to a male who is 1.75 m tall, weighs 70 kg, is 70 years old, and has a walk speed of 0.28 m⋅s^–1^. The experiments performed in the current study were designed for young males. Hence, the derived PT ranges in this study may have a limited capacity to assess thermal sensations or heat-related stress on elderly people. To improve thermal stress assessments for Koreans, further experiments that take into account the physiological responses of elderly people to thermal stress should be carried out.

Moreover, there are revealed significant differences between the genders for thermal comfort (Karjalainen 2012; Indraganti and Humphreys 2021). Females express more dissatisfaction than males in the same thermal environments (Karjalainen 2012). In particular, some studies indicated that elderly women are more affected by extreme heat and heat-related mortality than men (van Steen et al. 2019; Tong et al. 2014). However, assessing heat-related health risks more accurately for women is difficult because the current PT model was designed only for males. Therefore, further experiments that take into account the physiological responses of women to thermal stress should be carried out. Additionally, a new PT model for women should be developed to assess heat-related health risks more accurately for women. The derived PT range for various groups of people including women and elderly people could be determined through the experiments similar to the method described in this study.

## Conclusions

This study derived the Korean PT ranges of the thermal sensation classes related to heat stress based on the results of experiments using a controlled environmental chamber during summer in Seoul, South Korea. The PT ranges of thermal sensation classes were derived based on the TSV surveyed from the subjects. The Korean PT ranges were validated using data not used in the derivation and led to a better performance than the reference PT ranges for the assessment of thermal sensations of Koreans. While the reference PT ranges overestimated thermal sensations and thermo-physiological stress, particularly in the ‘Hot’ thermal sensation class, the Korean PT ranges provided robust predictions. The PT thresholds of ‘Warm,’ ‘Hot,’ and ‘Very hot’ thermal sensation classes for Koreans were 28 °C, 36 °C, and 43 °C, respectively. The values of the thresholds for Koreans were higher than those for Germans, which implies that the thermal sensations of heat environments experienced by Koreans and Germans differ. The relationship between PT and PMV for Germans is different from the relationship between PT and TSV by Koreans. The slope in the relationship between TSV and PT is lower than that in the relationship between PMV and PT. This result indicates that Koreans may have higher heat resistance or lower heat sensitivity than Germans. Because new PT ranges were derived based on experiments for young people, the predicted thermal sensations and thermal stresses may have a limited ability to assess heat-related health risks for the elderly people who is more vulnerable to heatwave events. To improve our ability to assess heat-related risk, the PT ranges for elderly models should be derived in a future study. Since PT can be used to assess cold stress, and as cold-related health risk is vital in assessing thermal health risks, the PT ranges of cold-related thermal sensations and stresses must also be derived based on experiments using the experimental chamber to assess the whole range of thermal stresses for Koreans using the PT.

## Supplementary Information

Below is the link to the electronic supplementary material.

Supplementary file1 (DOCX 16 KB)
