# Supplementary material for: Determination of thermal sensation levels for Koreans based on perceived temperature and climate chamber experiments with hot and humid settings
Source: Int J Biometeorol. 2022 Mar 4;66(6):1095–107. doi: 10.1007/s00484-022-02261-x (PMC9132799; doi:10.1007/s00484-022-02261-x)
Supplement: Supplementary file 1 — Supplementary file1 (DOCX 16 KB) [file 484_2022_2261_MOESM1_ESM.docx]

# Supplementary Information for: **Determination of thermal sensation levels for Koreans based on perceived temperature and climate chamber experiments with hot and humid settings**

Table S1. Information of used meteorological stations.

| No. | Name | Latitude (°) | Longitude (°) | Elevation (m) |
| --- | --- | --- | --- | --- |
| 1 | Sokcho | 38.251 | 128.565 | 18.06 |
| 2 | Chuncheon | 37.903 | 127.736 | 76.47 |
| 3 | Bukgangneung | 37.805 | 128.855 | 78.9 |
| 4 | Daegwallyeong | 37.677 | 128.718 | 772.57 |
| 5 | Seoul | 37.571 | 126.966 | 85.67 |
| 6 | Incheon | 37.478 | 126.625 | 68.99 |
| 7 | Wonju | 37.338 | 127.947 | 148.6 |
| 8 | Suwon | 37.272 | 126.985 | 34.84 |
| 9 | Uljin | 36.992 | 129.413 | 50 |
| 10 | Seosan | 36.777 | 126.494 | 28.91 |
| 11 | Cheongju | 36.639 | 127.441 | 58.7 |
| 12 | Andong | 36.573 | 128.707 | 140.1 |
| 13 | Daejeon | 36.372 | 127.372 | 68.94 |
| 14 | Pohang | 36.032 | 129.38 | 3.94 |
| 15 | Gunsan | 36.005 | 126.761 | 23.2 |
| 16 | Daegu | 35.878 | 128.653 | 53.5 |
| 17 | Jeonju | 35.841 | 127.119 | 61.4 |
| 18 | Ulsan | 35.583 | 129.335 | 82 |
| 19 | Gwangju | 35.173 | 126.892 | 72.38 |
| 20 | Jinju | 35.164 | 128.04 | 30.21 |
| 21 | Busan | 35.105 | 129.032 | 69.56 |
| 22 | Tongyeong | 34.845 | 128.436 | 32.3 |
| 23 | Mokpo | 34.817 | 126.381 | 38 |
| 24 | Yeosu | 34.739 | 127.741 | 64.64 |
| 25 | Wando | 34.396 | 126.702 | 35.24 |
| 26 | Jeju | 33.514 | 126.53 | 20.45 |
| 27 | Seogwipo | 33.246 | 126.565 | 49.03 |
